# 240. Transcriptional signatures differentiate pathogen- and treatment-specific host responses in patients with bacterial bloodstream infections

**DOI:** 10.1093/ofid/ofad500.313

**Published:** 2023-11-27

**Authors:** Joshua T Thaden, Felicia Ruffin, David Gjertson, Alexander Hoffmann, Vance G Fowler, Michael Yeaman

**Affiliations:** Duke University School of Medicine, Durham, North Carolina; Duke University Medical Center, Durham, North Carolina; University of California, Los Angeles, Los Angeles, California; University of California, Los Angeles, Los Angeles, California; Duke University Medical Center, Durham, North Carolina; UCLA, Torrance, CA

## Abstract

**Background:**

Clinical outcomes associated with bacterial bloodstream infections (BSI) are influenced by multiple factors, including the infecting bacterial species and choice of antibiotic therapy. However, the mechanisms by which differences in such factors influence patient outcomes are poorly understood. We hypothesized that variations in bacterial etiology and antibiotic therapy may alter clinical outcomes in part through differential host gene expression signatures. Therefore, we aimed to identify bacterial- and antibiotic-specific host transcriptional signatures in patients with bacterial BSI.

**Methods:**

RNA-Seq was performed on whole blood samples in patients infected with BSI due to prototypic Gram-negative vs. Gram-positive bacterial pathogens: *Escherichia coli* (n=30) or *Klebsiella pneumoniae* (n=28) vs. methicillin-susceptible *Staphylococcus aureus* [MSSA] (n=24) or methicillin-resistant *S. aureus* (MRSA) (n=58). Patients were matched by age, gender, and race. Differential gene expression and gene co-expression analyses were performed (Fig 1).

Figure 1

**Figure:**
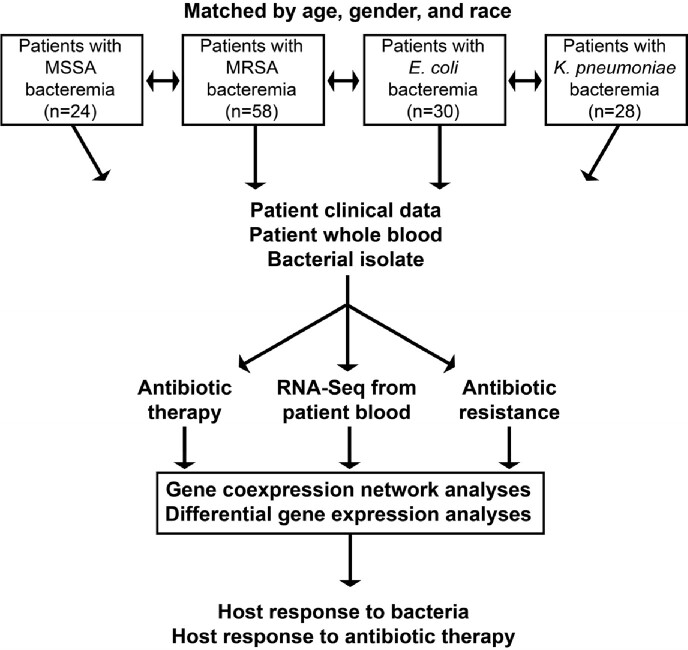

Overview of study design.

**Results:**

Clinical characteristics of patients with BSI in this study are shown in Table 1. No significant differences were detected in overall host transcriptomes in patients with *E. coli* versus *K. pneumoniae* BSI, so these groups were considered together as gram-negative BSI. Relative to *S. aureus* BSI, patients with gram-negative BSI had increased activation of the classical complement system (Fig 2). However, the most significant signal was a reduction in host transcriptional signatures involving mitochondrial energy transduction and oxidative burst in MRSA vs. MSSA (Fig 3). This attenuated host transcriptional signature in MRSA BSI remained after controlling for antibiotic therapy.

Table 1

**Figure:**
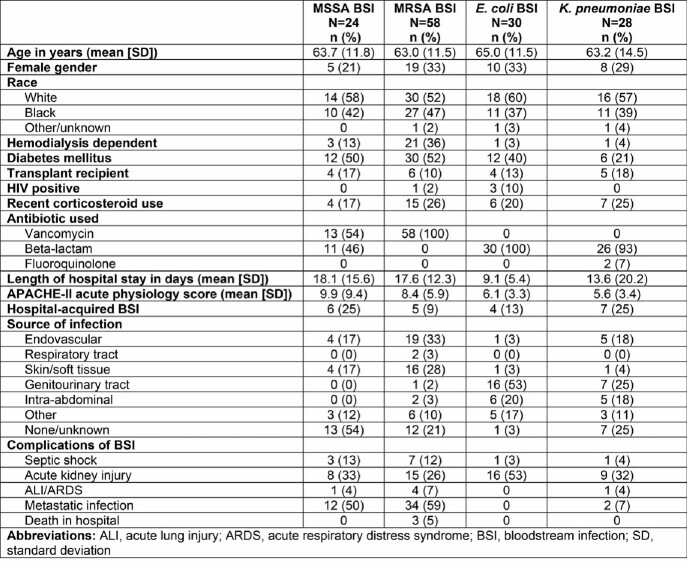

Demographics and outcomes of patients with bacterial bloodstream infections (BSI) included in this study.

Figure 2

**Figure:**
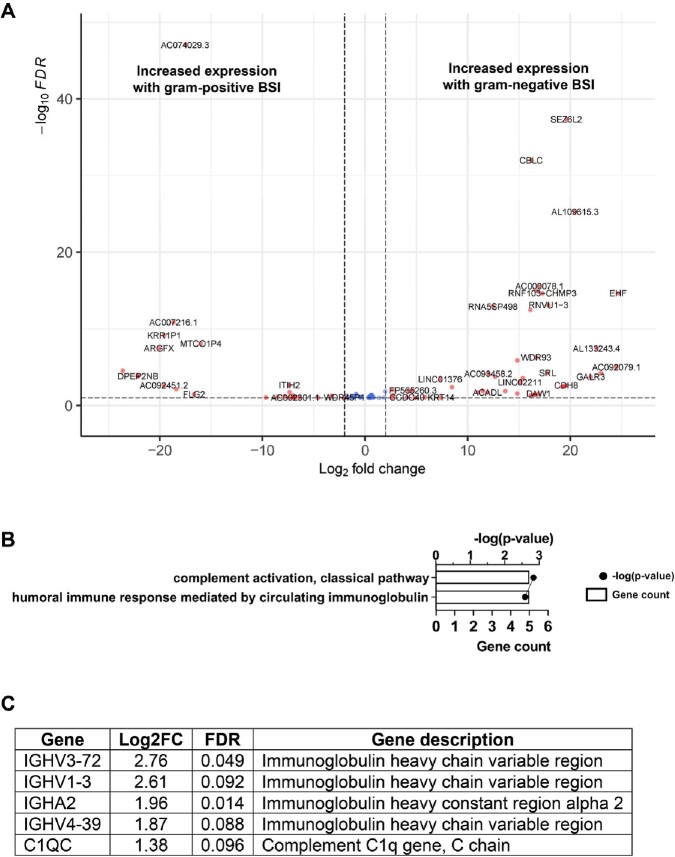

Whole blood gene transcript level changes in patients with gram-negative bloodstream infection (BSI) relative to gram-positive (i.e., S. aureus) BSI. (A) Volcano plot showing gene transcript level differences in patients with gram-negative versus S. aureus BSI. Genes that had log2-transformed fold change (Log2FC) greater than 1 or less than -1 and had a false discovery rate (FDR) less than 0.1 are shown in red. Genes meeting only the Log2FC or FDR criteria are shown in green and blue, respectively. Genes meeting neither criteria (NS) are shown in gray. (B) g:Profiler biological processes associated with the gene transcript differences in patients with gram-negative versus S. aureus BSI. For each biological process, the number of genes associated with the process (count) and p-value associated with the process are shown. In (C), the genes associated with the process shown in (B) are listed.

Figure 3

**Figure:**
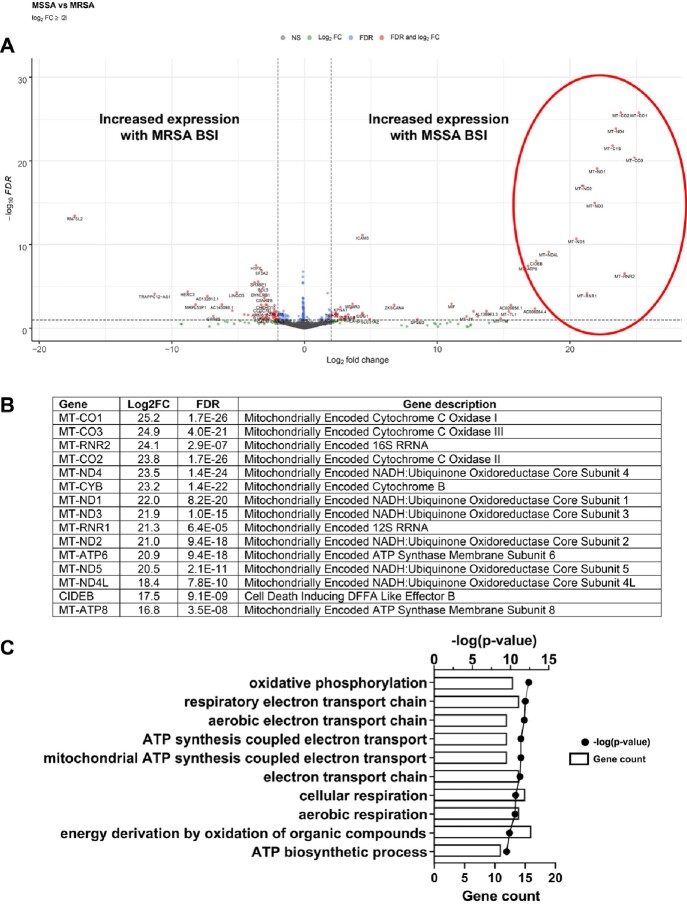

Whole blood gene transcript level changes in patients with methicillin-susceptible Staphylococcus aureus (MSSA) bloodstream infection (BSI) relative to methicillin-resistant S. aureus (MRSA) BSI. (A) Volcano plot showing gene transcript level differences in patients with MSSA versus MRSA BSI. Genes that had log2-transformed fold change (Log2FC) greater than 1 or less than -1 and had a false discovery rate (FDR) less than 0.1 are shown in red. Genes meeting only the Log2FC or FDR criteria are shown in green and blue, respectively. Genes meeting neither criteria (NS) are shown in gray. Circled in red is a group of genes that are particularly different between the two groups and further described in (B). In (B), the log2-transformed fold changes (Log2FC) (C) g:Profiler biological processes analysis of gene transcript level differences in patients with MSSA versus MRSA BSI. For each pathway, the number of genes associated with the pathway (count) and p-value associated with the pathway are shown.

**Conclusion:**

We uncovered an MRSA-specific host transcriptional signature that was driven in large part by suppressed mitochondrial responses necessary for essential immune functions in oxidative killing of *S. aureus*. Given the importance of respiratory functions and reactive oxygen species in eliminating intracellular *S. aureus,* this finding may offer new insights into persistence of MRSA BSI relative to other bacteria.

**Disclosures:**

**Joshua T. Thaden, MD, PhD**, Resonantia Diagnostics, Inc: Advisor/Consultant **Vance G. Fowler, MD, MHS**, Amphliphi Biosciences, Integrated Biotherapeutics; C3J, Armata, Valanbio; Akagera, Aridis, Roche, Astra Zeneca: Advisor/Consultant|Genentech, Regeneron, Deep Blue, Basilea, Janssen;: Grant/Research Support|Infectious Diseases Society of America: Honoraria|MedImmune, Allergan, Pfizer, Advanced Liquid Logics, Theravance, Novartis, Merck; Medical Biosurfaces; Locus; Affinergy; Contrafect; Karius;: Grant/Research Support|Novartis, Debiopharm, Genentech, Achaogen, Affinium, Medicines Co., MedImmune, Bayer, Basilea, Affinergy, Janssen, Contrafect, Regeneron, Destiny,: Advisor/Consultant|Sepsis diagnostic: Patent pending|UpToDate: Royalties|Valanbio and ArcBio: Stock Options

